# Effectiveness of electronic guideline-based implementation systems in ambulatory care settings - a systematic review

**DOI:** 10.1186/1748-5908-4-82

**Published:** 2009-12-30

**Authors:** Annemie Heselmans, Stijn Van de Velde, Peter Donceel, Bert Aertgeerts, Dirk Ramaekers

**Affiliations:** 1 School of Public Health, Katholieke Universiteit Leuven, Kapucijnenvoer 35 blok d, 3000 Leuven, Belgium; 2 Belgian Branch of the Cochrane Collaboration, Belgian Centre for Evidence-Based Medicine, Kapucijnenvoer 33 Blok j, 3000 Leuven, Belgium; 3 ZNA Hospital Network Antwerp, Leopoldstraat 26, 2000 Antwerp, Belgium

## Abstract

**Background:**

Electronic guideline-based decision support systems have been suggested to successfully deliver the knowledge embedded in clinical practice guidelines. A number of studies have already shown positive findings for decision support systems such as drug-dosing systems and computer-generated reminder systems for preventive care services.

**Methods:**

A systematic literature search (1990 to December 2008) of the English literature indexed in the Medline database, Embase, the Cochrane Central Register of Controlled Trials, and CRD (DARE, HTA and NHS EED databases) was conducted to identify evaluation studies of electronic multi-step guideline implementation systems in ambulatory care settings. Important inclusion criterions were the multidimensionality of the guideline (the guideline needed to consist of several aspects or steps) and real-time interaction with the system during consultation. Clinical decision support systems such as one-time reminders for preventive care for which positive findings were shown in earlier reviews were excluded. Two comparisons were considered: electronic multidimensional guidelines versus usual care (comparison one) and electronic multidimensional guidelines versus other guideline implementation methods (comparison two).

**Results:**

Twenty-seven publications were selected for analysis in this systematic review. Most designs were cluster randomized controlled trials investigating process outcomes more than patient outcomes. With success defined as at least 50% of the outcome variables being significant, none of the studies were successful in improving patient outcomes. Only seven of seventeen studies that investigated process outcomes showed improvements in process of care variables compared with the usual care group (comparison one). No incremental effect of the electronic implementation over the distribution of paper versions of the guideline was found, neither for the patient outcomes nor for the process outcomes (comparison two).

**Conclusions:**

There is little evidence at the moment for the effectiveness of an increasingly used and commercialised instrument such as electronic multidimensional guidelines. After more than a decade of development of numerous electronic systems, research on the most effective implementation strategy for this kind of guideline-based decision support systems is still lacking. This conclusion implies a considerable risk towards inappropriate investments in ineffective implementation interventions and in suboptimal care.

## Background

Physicians are encouraged to integrate the best available scientific evidence with clinical expertise and patient values in their routine medical practice. Because clinicians often do not have the time or the skills to retrieve and appraise the ever-increasing health evidence base, the evidence can be provided by instruments such as clinical practice guidelines (CPGs). However, the implementation of CPGs is often cumbersome, and a large number of randomized controlled trials (RCTs) and systematic reviews have already examined the cost-effectiveness of different guideline implementation strategies including electronic approaches [1,2].

Electronic guideline-based decision support systems have been suggested to successfully deliver the knowledge embedded in evidence-based guidelines to patients [3]. A number of studies have already shown positive findings for decision support systems, in areas such as drug-dosing systems and computer-based reminder systems for preventive care services [4,5]. An earlier review of Shiffman *et al*. [6] investigated the functionality and effectiveness of computer-based guideline implementation systems but this is now dated given the technological evolution and the burgeoning amount of computerized guidelines of the last decade. More recent systematic reviews of clinical decision support systems exist, but they assess a heterogeneous group of systems [7,8].

The objective of this systematic review was to systematically and comprehensively search the literature for studies evaluating the effectiveness of computer-based guideline implementation systems in ambulatory care settings with the multidimensionality of the guideline (the guideline needed to consist of several aspects or steps) and real-time interaction with the system during consultation as important inclusion criteria.

## Methods

### Selection criteria

An electronic guideline implementation method was defined as an electronic system directly supporting evidence-based clinical decision making in which point-of-care advice is provided based on one or more CPGs. The basic requirement to include an intervention in the systematic review was the electronic implementation of one or more multidimensional CPGs as a single intervention for physicians' use.

General expert systems and systems for education of healthcare professionals were not included in the review. Provider order entry systems were only included if they were accompanied by one or more electronic guidelines.

For the purpose of this study we defined a set of criteria to which the guideline system and the guideline had to correspond. Minimum criteria were:

1. The implemented guideline needed to consist of several aspects or steps. Brief prompts based on, *e.g*., simple age-related algorithms that could be electronic alerts for vaccination or screening were excluded. Dose calculation systems and alerts for drug-drug interactions were not included.

2. The development process of the implemented guideline needed to be transparent and well-documented. The development group was known and/or literature review was available.

3. Guidelines for prevention, as well as diagnosis, therapy, or management of a particular disease were included.

4. The mode of evidence delivery needed to be on-screen with system interaction during consultation. This criterion served to distinguish between recommendations presented to physicians on a computer screen from computer-generated output on paper, which was a reason for exclusion. The electronic recommendations had to be accessible during consultation, either automatically within the routinely used electronic medical records (EMRs) (*e.g*., via a pop-up screen) or on the initiative of the physician himself. Personal digital assistant systems were excluded.

System implementations supported by one or more additional interventions were included as long as the additional interventions concerned components of an implementation strategy, were of secondary importance, and were targeted at physicians.

Any study in which the main group of end users (>50%) consisted of physicians were included. Systems designed only for patients, nurses, dentists, pharmacists, physiotherapists, or other healthcare workers were not selected, nor were systems designed specifically for the treatment of hospitalised patients. The main focus was guideline implementation in outpatient medical care. Systems designed for a combined group of outpatients as well as inpatients also were excluded.

Two types of outcome measures were considered: patient outcomes with direct and surrogate endpoints, (*e.g*., blood pressure, blood glucose levels) and process outcomes such as physician adherence or compliance to CPGs, organisational, logistic, and financial issues. Quantitative outcome measures for which no comparison value existed in the control group, (*e.g*., use of the system or time using the system) were not selected.

Only hypothesis-testing studies in a real clinical environment based on a comparison between groups or across time periods were included in analysis *i.e*., RCTs, controlled clinical trials (CCT), controlled before-after studies (CBA), and interrupted time-series (ITS). RCTs with randomization at the level of the patient were excluded because of the major methodological flaw that the physicians were required to manage patients in the control and experimental groups concurrently.

### Search strategy

A literature search of the English literature (1990 to December 2008) indexed in the Medline database, Embase, the Cochrane Central Register of Controlled Trials and CRD (DARE, HTA and NHS EED databases) was conducted. The search strategy was sensitive. The search in OVID Medline was performed using either one of the following MeSH terms: decision support systems, clinical - decision making, computer-assisted - therapy, computer-assisted - drug therapy, computer-assisted - decision support techniques - computerized medical records systems - reminder systems - expert systems. Synonyms for 'guideline' were used as free text words in combination with the MeSH terms. The search string was adapted corresponding to the characteristics of the other electronic databases (detailed search strategy in appendix, see Additional File 1). The reference lists of all relevant studies and related systematic reviews were explored, Google scholar was searched to ensure that no studies were missed.

### Review process

Two reviewers independently selected studies from the titles and abstracts of all the retrieved references. Full texts of the remaining articles were then evaluated and irrelevant studies were excluded. The methodological quality was independently evaluated by two reviewers using the EPOC data collection checklists [9]. A study was judged as having a low risk of bias if all criteria were rated as done or not applicable; a moderate risk of bias was assigned if one or two criteria were not done, partially done, or not clear; and a high risk of bias was assigned if three or more criteria were not done, partially done, or not clear. Studies were excluded from final analysis summary in the case of major methodological flaws. Authors of selected studies were contacted if certain data were not reported in the article. If essential information could not be obtained, the study was excluded from further analysis. Relevant data of the remaining included studies were extracted by one reviewer and checked by another. Disagreements between assessors were discussed and resolved by consensus. In case of no consensus agreement, a third reviewer was consulted.

## Results

**Figure 1:**
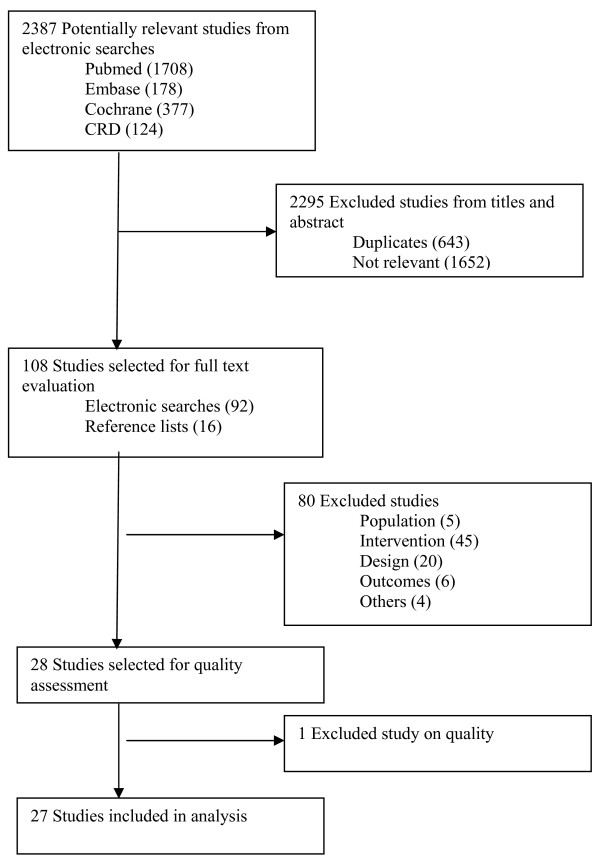
**Flowchart of identification and selection of studies**.

An initial electronic search yielded a total of 2,387 titles and abstracts of which 92 were judged to be potentially relevant based on title and abstract reading. Full texts of these 92 retrieved articles were reviewed. We finally selected 26 studies that met the inclusion criteria and rejected 66 studies. Another 16 articles were identified from reference searching of which one was included in final analysis. A flow chart through the different steps of study selection is provided in Figure 1. A Cohen's kappa of 0.82 was reached for inter-reviewer agreement.

From the 27 selected studies, there were twenty cluster RCTs, one CCT study, two CBA designs, and four ITS. The included studies are characterised by heterogeneity of clinical areas, format of interventions, and outcome parameters.

### Excluded studies

A total of 81 studies were rejected, five studies were excluded on population, 45 on intervention, 20 on design, six on outcome, one on methodological quality and four for other reasons. A table with references and reasons for exclusion can be found in Additional file 2.

### Included studies

#### Setting and participants

The majority of studies (52%) were conducted in the USA [10-23], four in the UK [24-28], five in the Netherlands [29-33], two in Norway [34,35], one in France [36], and one in Finland [37]. All studies evaluated the implementation of electronic guidelines in ambulatory care of which four were performed in the emergency department [18-20,36].

The studies of Hetlevik [34,35], Tierney[15,16], Murray[17], Schriger [19,20], and Day [18] each relate to evaluations of the same system across different modules. Because the modules were separately assessed as being independent systems, we considered them as different systems.

The number of professionals participating in the studies was difficult to determine because some of the studies reported in terms of the number of participating practices, others published the number of healthcare providers. The number of patients in the studies varied from a few hundred to a few thousand, ranging from paediatric to geriatric patients.

#### Intervention

Twenty-one studies concerned the implementation of guidelines for disease management, of which 71% provided support for chronic diseases [12-17,22-26,30,32,34,35], 24% for acute diseases [10,18-21] and one for both [37]. Because brief prompts were excluded, only one system was included that addressed screening and/or prevention [11]. Three studies assessed the effectiveness of electronic guidelines on radiology requests and test ordering [29,31,36], one was related to cancer genetics [27], and one to evidence-based prescribing [33]. The studies of Hetlevik [34,35], Rollman [13], Schriger [19,20], Day [18] and Jousimaa [37] were designed to assist in the diagnosis as well as management of the disease.

Targeted diseases were angina [24], coronary artery disease (CAD) [14], heart failure and ischemic heart disease [15], hypertension [17,22,26,34], dyslipidemia [32], hypercholesterolemia [33], diabetes [12,14,35], asthma/chronic obstructive pulmonary disease (COPD) [16,24,25,30,33], depression [13], HIV [23], occupational exposure to body fluids [19], acute low back pain [18], otitis media [21], and fever in children [20]. The study by Davis was related to various common pediatric diseases [10] and the study by Jousimaa [37] to various primary care problems. The system for prevention and screening was related to tobacco use cessation[11]. The intervention periods fluctuated around 12 months, ranging from four weeks [37] to 50 months (at one site in the study of Davis [10]).

The types of comparison were grouped as: electronic guidelines versus usual care [10-14,18-23,25,26,29,32-36] and electronic guidelines versus another guideline-based implementation method [13,15-17,24,26,27,30-32,37].

#### Outcome

A variety of outcome parameters were reported in each study, of which a vast majority investigated multiple outcomes of different data types. In the main process outcomes, such as guideline compliance, were addressed. The reporting of patient outcomes (*e.g*., blood pressure, cholesterol levels) was often complicated by methodological problems, and was limited.

### Risk of bias in included studies

The methodological quality of included studies was diverse. Several criteria could not be rated because of insufficient information.

We identified 20 cluster-RCTs [10,12-17,21,22,24-27,30-35,37], one CCT [23], and two CBA studies [11,29]. Reporting of specific procedures for concealment was considered less important because it concerned cluster-RCTs. The unit of randomization was the healthcare provider in five studies [10,12,13,21,37], physicians' practices in nine trials [22,24-27,30-33], health center or clinic in three studies [14,34,35], and practice session in another three trials [15-17]. The majority of studies accounted for clustering in their sample size calculations and their statistical techniques. Caution is needed with the interpretation of the results when clustering is not taken in account [11,23,29,34,35].

Most studies had similar baseline measurements for the primary outcomes or controlled for baseline imbalance in their statistical analyses [11-17,22,24,27,29,31,32,35,37]. A lack of information on this topic especially for the (primary) outcomes could potentially influence the results of the studies by McCowan, Kuilboer, Martens, and Safran [23,25,30,33].

The study of McCowan [25] possibly suffered from attrition bias because of insufficient follow-up. It was not possible to draw conclusions on this topic for the studies of Szpunar [11] and Sequist [14] because of insufficient data. An intention-to-treat analysis was performed and explicitly reported in eight trials [10,12,13,22,24,26,27,29].

Seven studies [11,22,25,26,30,34,35] scored negatively for the criterion 'blinded assessment of primary outcomes' Another source of bias may be the non-blinding of the healthcare providers. Because of the nature of the intervention, healthcare professionals could not be blinded to the intervention. Only the balanced incomplete block design of Eccles [24] and Martens [33] controlled for a possible Hawthorne effect. The reliability of the primary outcomes was scored positively in 11 studies [10,13-15,21,23,24,29,31-33].

As a result of the cluster design of most trials, the risk of intervention contamination could be minimised in all of included studies. Although minimal, completely eradicating this type of bias with this kind of intervention is nearly impossible for most of the studies.

Four ITS designs [18-20,36] were included. Three of these ITS designs consisted of the analysis of different modules of one and the same system [18-20]. The study of Carton [36] was the only study with sufficient data points before and after the intervention and where data collection methods were identical before and after the intervention. It was not possible to draw definite conclusions on the completeness of data sets for the study of Carton [36], for the three other studies [18-20] the data sets were incomplete. The reasons for the number of points pre and post intervention were not given in any of the studies and none of the studies provided information on the shape of the intervention effect. Data were analysed appropriately in the studies by Schriger [19,20] and Day [18]. 'Protection against secular changes' was only explicitly reported in the study by Schriger [19]. Outcomes were assessed blindly and measured reliable in all of the studies. Because of insufficient essential methodological information, all ITS designs were judged to be at a high risk of bias.

### Effects of intervention

A meta-analysis was not performed due to the risk of bias in some of included studies and the heterogeneity in outcome measures. We were unable to calculate the corrected odds ratios for the individual studies because we could not correct for clustering. Because of this, we had to rely on the effect sizes reported in the articles, which could be biased in studies which had methodological flaws. Summaries of findings are reported in Tables 1, 2 and 3. An intervention was defined as successful when at least 50% of the outcomes were statistically significant (alpha = 0.05 (without correction for multiple comparisons in a lot of studies)).

**Table 1:** Summary of Findings for comparison one: Electronic multidimensional guidelines versus usual care

Study	Risk of bias	No of patients/professionals	Intervention	Outcomes	
				
				Process	Patient
**Christakis **[21] Cluster-RCT	low	1,339 visits for OM, 38 physicians	Evidence-based (EB) message system presenting real time evidence to providers based on their prescribing practice for otitis media (OM).	Y	

**Davis **[10] Cluster-RCT	low	12,195 visits, 44 healthcare providers	EB message system that presented real-time evidence to providers based on prescribing practices for acute otitis media, allergic rhinitis, sinusitis, constipation, pharyngitis, croup, urticaria, and bronchiolitis	Y	

**Meigs **[12] Cluster-RCT	moderate	598 patients, 66 healthcare providers	Diabetes management application, (DMA); interactive patient-specific clinical data, treatment advice, and links to other web-based resources	N	N

**Montgomery **[26] Cluster-RCT	moderate	614 patients, 27 general practices	Computer-based clinical decision support system and a risk chart on absolute cardiovascular risk, blood pressure, and prescribing of cardiovascular drugs in hypertensive patients.	N	N

**Rollman **[13] Cluster-RCT	moderate	200 patients, 17 primary care physicians	Guideline-based treatment advice for depression Active care Passive care	N N	N N

**van Wyk **[32] Cluster-RCT	moderate	87,866 patients, 77 primary care physicians	Clinical decision support system with respect to screening and treatment of dyslipidemia Alerting version On-demand version	Y N	

**Carton **[36] ITS design	high	6,869 radiological examinations in sample	Reminder on screen, indicating the appropriate recommendations concerning radiology requests	N	

**Day **[18] ITS design	high	off: 103 patients on: 258 patients off: 125 patients	Real-time advice regarding documentation, testing, treatment and disposition of emergency department patients with low back pain (EDECS)	N	

**Hetlevik '99 **[34] Cluster-RCT	high	2,230 patients, 53 physicians	Clinical decision support system for hypertension		N

**Hetlevik '00 **[35] Cluster-RCT	high	1,034 patients, 53 physicians	Clinical decision support system for diabetes mellitus		N

**Hicks **[22] Cluster-RCT	high	2,027 patients, 14 primary care practices	Electronic decision support for hypertensive patients	Y	N

**McCowan **[25] Cluster-RCT	high	477 patients, 17 practices	Computerized decision support system for the management of asthma	N	N

**Poley **[29] CBA design	high	109 primary care physicians	Guideline-driven decision-support system for ordering blood tests in primary care	N	

**Safran **[23] CCT	high	349 patients in analysis, 126 physicians and nurses	Reminders and alerts for HIV infection	Y	N

**Schriger '97 **[19] ITS design	high	off: 50 patients on: 156 patients off: 74 patients	Real-time advice regarding documentation, testing, treatment and disposition of emergency department patients regarding the management of body fluid exposure (EDECS)	Y	

**Schriger '00 **[20] ITS design	high	off: 352 patients on: 374 patients off: 104 patients	Real-time advice regarding documentation, testing and treatment of children with fever presenting in the emergency department (EDECS)	N	

**Sequist **[14] Cluster-RCT	high	Diabetes: 4,549 patients - CAD: 2,199 patients, 194 physicians	EB electronic reminders for diabetes and coronary artery disease (CAD)	N	

**Szpunar **[11] CBA design	high	Pre: 5,334 patients - Post: 3,970 patients, 6 clinics	Tobacco Use Cessation (TUC) Automated Clinical Practice Guideline	Y	

**Martens **[33] Cluster-RCT	high	53 primary care physicians	Intervention group one: Reminders on antibiotics, asthma/chronic obstructive pulmonary disease (COPD) Intervention group two: Reminders on cholesterol-lowering drugs	N	

Y = if at least 50% of outcomes significant, N = if less than 50% of outcomes significant

**Table 2:** Summary of Findings for comparison two: Electronic guideline implementations versus paper version of the guideline

Study	Risk of bias	No of patients/professionals	Intervention	Outcomes	
				
				Process	Patient
**Eccles **[24] Cluster-RCT	low	2,400 patients, 60 primary care practices	Intervention group one: Computerized asthma guidelines + paper version of the guidelines for asthma and angina Intervention group two: Computerized angina guidelines + paper version of the guidelines for asthma and angina	N	

**Tierney '03 **[15] Cluster-RCT	low	706 patients, 246 physicians	Intervention group one: Computerized cardiac care suggestions + printed summary of the guidelines Intervention group two and three: not included in analysis of this review Control group: Usual care + printed summary of the guidelines	N	N

**Tierney '05 **[16] Cluster-RCT	low	706 patients, 246 physicians	Intervention group one: Computerized feedback for asthma and COPD + printed summary of the guidelines Intervention group two and three: not included in analysis of this review Control group: Usual care + printed summary of the guidelines	N	N

**Jousimaa **[37] Cluster-RCT	moderate	2,813 evaluated cases, 130 physicians	Intervention group: CD ROM of primary care guidelines Control group: Text based version of primary care guidelines	N	

**Montgomery **[26] Cluster-RCT	moderate	614 patients, 27 primary care practices	Intervention group: Computer-based clinical decision support system and a risk chart on absolute cardiovascular risk, blood pressure, and prescribing of cardiovascular drugs in hypertensive patients. Control group: Cardiovascular risk chart on paper alone	N	N

**Murray **[17] Cluster-RCT	moderate	712 patients, 246 physicians	Intervention group one: Computerized suggestions for hypertension + printed, referenced summary of the locally approved guidelines Intervention group two and three: not included in analysis of this review Control group: Usual care + printed, referenced summary of the locally approved guidelines	N	N

**Wilson **[27] Cluster-RCT	moderate	86 practices	Intervention group: Electronic referral guidelines for breast cancer + mailed referral guidelines Control group: Usual care + mailed referral guidelines	N	N

**Kuilboer **[30] Cluster-RCT	high	156,772 patients, 40 primary care physicians	Intervention group: AsthmaCritic provides patient-specific feedback for asthma and COPD + disposal of the asthma and COPD guidelines Control group: Usual care + disposal of the asthma and COPD guideline	N	

Y = if at least 50% of outcomes significant, N = if less than 50% of outcomes significant

**Table 3:** Summary of Findings for comparison two: Comparison of different types of electronic guideline implementation

Study	Risk of bias	No or patients/professionals	Intervention	Outcomes	
				
				Process	Patient
**Van Wijk **[31] Cluster-RCT	low	7,094 patients, 44 primary care practices	Intervention group one: BloodLink Guideline Intervention group two: BloodLink Restricted	Y	
				
				In favour of BloodLink Guideline	

**Rollman **[13] Cluster-RCT	moderate	200 patients, 17 primary care physicians	Intervention group one: Guideline-based treatment advice for depression: active care Intervention group two: Guideline-based treatment advice for depression: passive care	N	N

**Van Wyk **[32] Cluster-RCT	moderate	87,866 patients, 77 primary care physicians	Intervention group one: Clinical decision support system with respect to screening and treatment of dyslipidemia: alerting version Intervention group two: Clinical decision support system with respect to screening and treatment of dyslipidemia: on-demand version	Y	
				
				In favour of the alerting version	

Y = if at least 50% of outcomes significant, N = if less than 50% of outcomes significant

### Comparison one: Electronic multidimensional guidelines versus usual care

Nineteen studies-- twelve cluster-RCTs, one CCT, two CBA trials and four ITS designs--assessed the effectiveness of electronic guideline implementation systems compared with a usual care control group [10-14,18-23,25,26,29,32-36]. The majority investigated only process outcomes and reported at least one statistically significant process outcome in favour of the intervention group. However, in most trials this finding was not consistent throughout the study, and the authors were not unanimous in endorsing the effectiveness of electronic guidelines.

None of the studies in comparison one showed better patient outcomes, and seven of seventeen studies that investigated process showed improvements in process of care variables.

A subgroup analysis was performed to identify potential guideline or system characteristics that could predict success. Odds ratios for the following subgroups were determined: local guidelines versus national guidelines, advice alone versus advice plus a link to the evidence or the full text of the guideline, automated advice versus having to actively seek it, type of targeted decision (test ordering, therapy, diagnosis or diagnosis plus therapy), integrated into EMR versus not integrated. None of the odds ratios was statistically significant at a significance level of p < 0.05.

Reported reasons for failure to show an effect were work overload and time pressure [12-15,25,35], low levels of use of the system [12,24,25,35], lack of integration within the normal workflow [12,14], lack of patient participation [13,24,35], technical problems [25], controversy about the implemented guideline [15,18,20], or highly complex suggestions [17].

A summary of the results of the studies in comparison group one is given in Table 1. An expanded table of the results can be found in Additional file 3.

### Comparison two: Electronic multidimensional guidelines versus another guideline implementation method

Eleven studies were available for this comparison. Systems were classified into two groups. One group investigated the differences in effect between the electronic implementation of the guideline and the distribution of a paper version of the guideline, and the other group assessed the differences in effect between two different types of electronic implementation. Eight studies were included in the analysis of the first group [15-17,24,26,27,30,37] and three in the second group [13,31,32]. We could not find an incremental effect of the electronic implementation over the distribution of paper versions of the guideline. None of the studies showed better patient or process outcomes in favour of the electronic implementation. The only significant difference was found in the study by Montgomery [26] where the computer support group fared worse, having a poorer cardiovascular risk reduction than the chart-only group.

The variability in the different types of electronic implementation and the limited amount of studies in the second group made it impossible to reach a firm conclusion concerning the success of a specific type of electronic implementation. The conclusions of the authors of the studies are summarized in Table 2 and 3. An expanded table of the results can be found in Additional file 3.

## Discussion

### Summary of main results

Our search yielded a limited number of studies investigating the effects of electronically implemented multidimensional guidelines.

The methodological quality of the included studies was variable. The majority of studies were cluster-RCT. Despite the clustering, most (75%) of the studies appropriately accounted for the clustered nature of the study data, if needed.

Most of included studies were designed to study a large variety of process outcomes and were inconsistent in their published results. Patient outcomes were not widely studied. It is important to consider whether the proper goal of decision-support systems is to improve process outcomes (such as provider performance) or patient outcomes [38-40]. Patient outcomes are not easily measured [41]; they often rely on patient compliance and severity of the disease and generally require longer periods of assessment. Many researchers would agree that showing an improvement in a process measure based on good evidence is sufficient. We acknowledge that process of care endpoints can be very appropriate. But because the purpose of many guidelines is to improve patient care, we believe that patient outcomes should be measured more often, or that at least the link between process indicator and final patient outcome should have been validated.

We found no evidence of an effect on patient outcomes, but the evidence is more mixed in terms of process of care. With success defined as at least 50% of the outcome variables being significant, none of the studies were successful in improving patient outcomes. Only seven of seventeen studies that investigated process outcomes showed improvements in process of care variables compared with the usual care group (comparison one). No incremental effect of the electronic implementation over the distribution of paper versions of the guideline was found, for either patient outcomes or the process outcomes (comparison two). Although there is a risk of studies selectively reporting only positive results [42], we do not think that this is a major issue in this review because the results do not suggest a positive effect from electronic guideline-based implementation systems.

The review by Kawamoto *et al*. [7] report four predictors of improved clinical practice using clinical decision support systems. Two of them were used as inclusion criteria for this review, namely 'provision of decision support at the time and place of consultation' and 'computer-based decision support'. Despite this promising starting point, we could not find sufficient scientific evidence to support the widespread implementation of complex electronic guideline systems at the moment. It should be stressed that the exclusion of computer-generated paper output and the electronic implementations of single-step guidelines and alert systems probably had an important effect on our main finding. In contrast to the results of our review, computer-based simple reminders have been demonstrated to be effective in increasing physicians' compliance, for a single procedure [4]. A meta-analysis by Shea *et al*. [5] supported the effectiveness of computer-based reminder systems to improve prevention services.

No uniform approach could be recognised in the functional or technical designs of the systems. The results of this review confirm the statement of James [43] that computerized CPGs must present the right information, in the right format, at the right time without requiring special effort. Tedious additional data entry, an overwhelming amount of feedback [15,35], software or hardware problems [25] were more than once reported as a key element for failure of the system. It is important that the data systems require are drawn from existing sources such as EMRs and that the system is as integrated into the entire workflow and possibly within the existing provider order entry system [12,14]. Ease, speed, and some control in the use of the system [44] seemed to be critical success factors according the Discussion section of several included studies [10,45], though further literature review to explore these topics was not done.

Collaboration with end-users [46] during the development process is essential in the design of the system, as is managerial support. Allowing end-users to identify their information needs for electronic implementation and the manner in which they would like to receive the recommendations seems to be one step in the right direction [15,47]. The possibility for updating the evidence and adding local practice-based evidence should be considered [48]. It is likely that an electronic guideline will lose its value with obsolete evidence not adapted to local practice.

### Overall completeness and applicability of the evidence

The evidence found is probably of limited generalisability. No guarantee exists that an electronic guideline which works in one setting will do the same in another [49], *e.g*., results of academic practice with its specific setting and characteristics may be different from everyday practice. The characteristics of the healthcare system in each country (*e.g*., financing systems) may also be important when generalising the results.

All included studies investigated possible benefits. None of them extensively explored potential harms, unless the outcomes could be interpreted as reversed benefits in favour of the control group. The surveys accompanying some trials and the discussion section of the studies sometimes made it possible to deduce some negative implications of the systems. As important as the potential harms is the duration of effect of a system after an extended period of time, which remains understudied at this time.

### Potential biases in the review process

The review process could possibly be influenced by two forms of bias. One form of potential bias is situated in the identification of potentially relevant studies, the other in the final selection of studies. Electronic guideline systems generating patient-specific reports on paper could have been excluded when the existence of simultaneous online recommendations was not sufficiently stressed. Our search strategy was chosen to be deliberately broad, but even more search terms that focused on the technical aspects could have been added. A manual search was not performed because it was not possible to determine a set of objective criteria for inclusion of one journal and exclusion of the other.

The lack of studies in this field could potentially be explained by the methodological difficulties that arise for this type of implementation research. A large number of narrative articles--often opinions or technical descriptions--and studies without control group can be found. In general, these publications advocate the use of electronic systems, and some of them have a commercial conflict of interest.

### Agreements with other studies or reviews

Although still heterogeneous concerning content and system design, the main difference between this systematic review and earlier reviews is the basic requirement of implementation of a guideline, on screen, at time and place of consultation, and the restriction in scope to multidimensional multi-step guidelines and to electronic systems in ambulatory care settings. This review seems to be an update of the review by Shiffman *et al*. [6], but included different studies and technologies. Most of included studies in the review by Shiffman were related to generating paper-based output by a computer, while this was used as an explicit exclusion criterion in our review. Shiffman *et al *[6] reported a guideline adherence and documentation improvement in fourteen of eighteen and four of four studies, respectively.

Other reviews relevant for this topic studied the effectiveness and/or efficiency of guideline dissemination and implementation strategies in general or discussed a heterogeneous group of (computerized) clinical decision support systems [4,7,8,46,50]. Garg *et al*. [8] updated earlier reviews by Johnston *et al*. [4] and Hunt *et al*. [46] investigating the effect of clinical decision support systems. All three reviews reported that computer-based decision support can improve clinical performance, the effects on patient health outcomes remained understudied and, when studied, inconsistent. These results were in line with the conclusions of Kawamoto *et al*. [7], who found a significant improvement in clinical practice in 68% of the trials. Cramer *et al*. [50] found the use of evidence delivery systems to enhance the process of care, but could not detect any effect on patient outcomes when pooling the data of the studies. A recent systematic review by Bryan *et al*. [51] studied the effectiveness of clinical decision support tools in ambulatory/primary care and concluded that clinical decision support tools (CDSS) have the potential to produce statistically significant improvements in outcomes: 76% of studies found either positive or variable outcomes related to CDSS intervention with 24% showing no significant effect.

Outcomes were not consistent throughout all trials in earlier reviews, and patient outcomes were seldom studied, which is in line with the results of our review. However, conclusions were, in general, more positive than the main findings of our review, probably due to the exclusion of the more effective simple reminder systems and computerized paper-generated output and the differences in the definition of a successful intervention. For example, the review by Bryan [51] classified studies with variable outcomes separately while the same studies could be classified as unsuccessful in our review when less than 50% of outcomes were statistically significant.

## Summary

There is little evidence at the moment for the effectiveness of an increasingly used and commercialised instrument such as electronic multidimensional guidelines. After more than a decade of development of numerous electronic systems, evidence on the most effective implementation strategy for this kind of guideline-based decision support systems is still lacking. This conclusion suggests the risk of inappropriate investments in ineffective implementation interventions and in suboptimal care. It is remarkable that healthcare payers require evidence on effectiveness and safety for other healthcare interventions, such as drugs and devices, and seem not to be concerned about the cost-effectiveness of organisational interventions such as electronic implementation of multidimensional CPGs that can also seriously impact patient care.

Future developments of this kind of information systems should incorporate a high-quality research design, and patient outcomes in concordance with other studies need to be studied. Not only studies investigating the benefits but also studies exploring potential harms, lasting effects, and both direct and indirect costs are important.

## Competing interests

The authors declare that they have no competing interests.

## Authors' contributions

All authors were involved in various stages of the study design. AH implemented and wrote the review. SVDV was the second reviewer and screened retrieved papers against inclusion criteria, appraised the methodological quality of studies, and checked if data extraction was accurate. DR supervised the study. DR, BA, and PD gave methodological advice and commented on subsequent drafts of the paper. All authors read and approved the final manuscript.

## Supplementary Material

Additional file 1 **Search strategy (OVID Medline)**. Search strategy performed in Medline. Click here for file

Additional file 2 **List of excluded studies**. List of excluded studies based on full text evaluation. Click here for file

Additional file 3 **Summary of Findings--expanded tables**. Expanded versions of the tables with the summary of findings. Table 1 for comparison one, electronic multidimensional guidelines versus usual care and Table 2 and 3 for comparison two, electronic multidimensional guidelines versus another guideline implementation method. Click here for file
