# Supplementary material for: Effectiveness of electronic guideline-based implementation systems in ambulatory care settings - a systematic review
Source: Implement Sci. 2009 Dec 30;4:82. doi: 10.1186/1748-5908-4-82 (PMC2806389; doi:10.1186/1748-5908-4-82)
Supplement: Additional file 1 — Search strategy (OVID Medline). Search strategy performed in Medline. [file 1748-5908-4-82-S1.DOC]

**Search strategy** (OVID Medline)

1/ Decision Support Systems, Clinical: focus

2/ Decision Making, Computer-Assisted: focus

3/ Therapy, computer-assisted: focus

4/ Drug therapy, computer-assisted: focus

5/ Decision support techniques: focus

6/ Expert Systems: focus

7/ Reminder Systems: focus

8/ Computerized medical records systems: focus

9/ 1 OR 2 OR 3 OR 4 OR 5 OR 6 OR 7 OR 8

10/ (protocol* or guideline* or guidance or recommendation* or reminder* or alert* or suggestion* or evidence or messag* or (decision adj0 support) or feedback or prompt* or intervention or implement* or prescri*).ti

11/ 9 AND 10

12/ limit 11 to (yr="1990 - 2008" and (clinical trial or comparative study or controlled clinical trial or evaluation studies or multicenter study or randomized controlled trial or research support, nih, extramural or research support, nih, intramural or research support, non us gov't or research support, us gov't, non phs or research support, us gov't, phs))
