# Supplementary material for: Effectiveness of electronic guideline-based implementation systems in ambulatory care settings - a systematic review
Source: Implement Sci. 2009 Dec 30;4:82. doi: 10.1186/1748-5908-4-82 (PMC2806389; doi:10.1186/1748-5908-4-82)
Supplement: Additional file 2 — List of excluded studies. List of excluded studies based on full text evaluation. [file 1748-5908-4-82-S2.DOC]

| List of excluded studies based on full text evaluation | | |
| --- | --- | --- |
| **Study** | **Type of exclusion** | **Reason for exclusion** |
| Agrawal [1] | Study design | No control group |
| Apkon [2] | Intervention | No multidimensional guideline |
| Augstein [3] | Intervention | KADIS decision support report, KADIS is based on a mathematical model that describes the glucose/insulin metabolism in type 1 diabetes in the form of a coupled differential equation system |
| Bassa [4] | Study design | Before-after study without control |
| Bindels [5] | Outcomes | Not studying relevant outcomes for our review, drop out of 9 of the 11 (82%) participating GP's in one cluster |
| Bindels [6] | Outcomes | Outcomes not relevant for our review |
| Bloomfield [7] | Intervention | No multidimensional guidelines |
| Bouaud [8] | Study design | Before-after study without control |
| Buising [9] | Intervention | Multifaceted intervention |
| Cannon [10] | Population | Research participants: One was a clinical psychologist, one was a registered nurse, one was a social worker, and one was an addiction therapist. |
| Cleveringa [11] | Intervention | Multifaceted intervention |
| Cobos [12] | Intervention | Intervention not only towards physicians, also promotion of healthy cardiovascular lifestyles towards patients |
| Dean [13] | Intervention | Not on-screen during consultation, form providing decision support for determining site of care and antibiotic selection.> form was faxed |
| Demakis [14] | Intervention | No multi-step guideline, different kind of alerts |
| Eccles [15] | Intervention | Not on-screen during consultation, messages were attached to the reports of the radiograph |
| Emery [16] | Intervention | Cancer risk assessment |
| Etter [17] | Population | Intervention towards patients |
| Feldstein [18] | Intervention | E-mail with link to internal and external resources, not on-screen during consultation |
| Filippi [19] | Intervention | Alert system |
| Fox [20] | Study design | Descriptive |
| Fung [21] | Study design | Test of the applicability of the computerized templates in a laboratory setting |
| Frances [22] | Intervention | Computerized and written reminders: mixed effects; insufficient data |
| Fretheim [23] | Intervention | Multifaceted intervention |
| Glasgow [24] | Intervention | Printouts for physicians, intervention mainly towards patients |
| Goergen [25] | Study design | Before-after study without control |
| Goldberg [26] | Intervention | Not on screen during consultation, information stored and displayed the following day |
| Goldberg [27] | Others | Duplicate of Goldberg et al 2000 |
| Harpole [28] | Population | In-patients’ orders |
| Hobbs [29] | Methodological quality | Analysis based on 14/25 practices + 5 practices did not enter any data in the system |
| Holbrook [30] | Intervention | Multifaceted intervention, part of intervention towards patients |
| Horowitz [31] | Intervention | Not electronic |
| Johnson [32] | Population | Intervention towards patients |
| Johnson [33] | Study design | Descriptive |
| Kashner [34] | Intervention | Not electronic |
| Keeffe [35] | Study design | Qualitative part of a RCT |
| Kenealy [36] | Intervention | Alert system |
| Khoury [37] | Intervention | Multifaceted intervention |
| Kitahata [38] | Study design | Before-after study without control |
| Lesourd [39] | Intervention | No guidelines, pregnancy rate of system vs pregnancy rate achieved by physicians |
| Lester [40] | Intervention | Via e-mail |
| Levenson [41] | Study design | Before-after study without control |
| Litzelman [42] | Intervention | Alerts |
| Lobach [43] | Intervention | On paper |
| Lobach [44] | Intervention | On paper |
| Lobach [45] | Intervention | On paper |
| Lorenzoni [46] | Study design | No control group |
| McCartney [47] | Intervention | No electronic guideline-based implementation system |
| McEwen [48] | Outcomes | Independent predictors of giving advice to quit smoking |
| McMullin [49] | Study design | Retrospective cohort study with artificial construction of a control group |
| Mitchell [50] | Intervention | No electronic guideline-based implementation system |
| Montori [51] | Intervention | Multifaceted intervention |
| Margolis [52] | Others | Discontinued after 5 weeks |
| Morgan [53] | Intervention | Alerts, (paper-based as well as on-screen) |
| Nease [54] | Intervention | Alerts + reminders were automatically printed in advance of patient appointments |
| Nease [55] | Study design | Observational design |
| Nilasena [56] | Intervention | Printed paper health maintenance report |
| Ornstein [57] | Intervention | Multifaceted intervention |
| Palchuk [58] | Study design | Descriptive |
| Pestotnik [59] | Population | In-patients + descriptive epidemiologic study |
| Plaza [60] | Others | Language: Spanish |
| Ramnarayan [61] | Study design | Simulated cases, not tested in a real clinical environment |
| Riddell [62] | Outcomes | Maori/non-Maori differences |
| Rios [63] | Study design | Observational design |
| Rossi [64] | Intervention | 1-page guideline reminder placed in the patient's chart |
| Roumie [65] | Intervention | Multifaceted intervention |
| Safran [66] | Others | Not found |
| Seroussi [67] | Study design | Before-after study without control |
| Shiffman [68] | Intervention | Handheld computers |
| Smith [69] | Intervention | Report on paper |
| Subramanian [70] | Intervention | Every night, the guideline program produced both Intervention and Control HF care suggestion reports for each enrolled patient who had a primary care appointment the following day. Reports were clipped to the patients’ outpatient charts |
| Thomas [71] | Intervention | Computer-generated report, patients had to complete a computer version of the CIS-R before consultation |
| Tolman [72] | Intervention | Dose calculation system |
| Toth-Pal [73] | Intervention | Alert system |
| van Steenkiste [74] | Intervention | Intervention also towards patients, mixed effects |
| van Wyk [75] | Study design | Descriptive |
| Vissers [76] | Outcomes | Outcomes not relevant for analysis in review |
| Vissers [77] | Outcomes | Outcomes not relevant for analysis in review |
| Wells [78] | Study design | Before-after study without control |
| Westfall [79] | Intervention | Report, no electronic guideline-based implementation system |
| Whitley [80] | Study design | Retrospective analysis |
| Ziemer [81] | Intervention | The patient-specific reminder was printed out and attached to the front of the chart each time a patient presents for a visit |

Reference List

1. Agrawal A, Mayo-Smith MF: **Adherence to computerized clinical reminders in a large healthcare delivery network.** *Stud Health Technol Inform* 2004, **107:**111-114.

2. Apkon M, Mattera JA, Lin Z, Herrin J, Bradley EH, Carbone M, Holmboe ES, Gross CP, Selter JG, Rich AS, Krumholz HM: **A randomized outpatient trial of a decision-support information technology tool.** *Arch Intern Med* 2005, **165:**2388-2394.

3. Augstein P, Vogt L, Kohnert KD, Freyse EJ, Heinke P, Salzsieder E: **Outpatient assessment of Karlsburg Diabetes Management System-based decision support.** *Diabetes Care* 2007, **30:**1704-1708.

4. Bassa A, del Val M, Cobos A, Torremade E, Bergonon S, Crespo C, Brosa M, Munio S, Espinosa C: **Impact of a clinical decision support system on the management of patients with hypercholesterolemia in the primary healthcare setting.** *Dis Manag Health Outcomes* 2005, **13:**65-72.

5. Bindels R, Hasman A, van Wersch JW, Talmon J, Winkens RA: **Evaluation of an automated test ordering and feedback system for general practitioners in daily practice.** *Int J Med Inform* 2004, **73:**705-712.

6. Bindels R, Hasman A, Derickx M, van Wersch JW, Winkens RA: **User satisfaction with a real-time automated feedback system for general practitioners: a quantitative and qualitative study.** *Int J Qual Health Care* 2003, **15:**501-508.

7. Bloomfield HE, Nelson DB, van Ryn M, Neil BJ, Koets NJ, Basile JN, Samaha FF, Kaul R, Mehta JL, Bouland D: **A trial of education, prompts, and opinion leaders to improve prescription of lipid modifying therapy by primary care physicians for patients with ischemic heart disease.** *Qual Saf Health Care* 2005, **14:**258-263.

8. Bouaud J, Seroussi B, Antoine EC, Zelek L, Spielmann M: **A before-after study using OncoDoc, a guideline-based decision support-system on breast cancer management: impact upon physician prescribing behaviour.** *Stud Health Technol Inform* 2001, **84:**420-424.

9. Buising KL, Thursky KA, Black JF, MacGregor L, Street AC, Kennedy MP, Brown GV: **Improving antibiotic prescribing for adults with community acquired pneumonia: Does a computerised decision support system achieve more than academic detailing alone?--A time series analysis.** *BMC Med Inform Decis Mak* 2008, **8:**35.

10. Cannon DS, Allen SN: **A comparison of the effects of computer and manual reminders on compliance with a mental health clinical practice guideline.** *J Am Med Inform Assoc* 2000, **7:**196-203.

11. Cleveringa FGW, Gorter KJ, Van Donk MD, Rutten GE: **Combined task delegation, computerized decision support, and feedback improve cardiovascular risk for type 2 diabetic patients.** *Diabetes Care* 2008, **31:**2273-2275.

12. Cobos A, Vilaseca J, Asenjo C, Pedro-Botet J, Sanchez E, Val A, Torremade E, Espinosa C, Bergonon S: **Cost effectiveness of a clinical decision support system based on the recommendations of the European Society of Cardiology and other societies for the management of hypercholesterolemia: Report of a cluster-randomized trial.** *Dis Manag Health Outcomes* 2005, **13:**421-432.

13. Dean NC, Suchyta MR, Bateman KA, Aronsky D, Hadlock CJ: **Implementation of admission decision support for community-acquired pneumonia.** *Chest* 2000, **117:**1368-1377.

14. Demakis JG, Beauchamp C, Cull WL, Denwood R, Eisen SA, Lofgren R, Nichol K, Woolliscroft J, Henderson WG: **Improving residents' compliance with standards of ambulatory care: results from the VA Cooperative Study on Computerized Reminders.** *JAMA* 2000, **284:**1411-1416.

15. Eccles M, Steen N, Grimshaw J, Thomas L, McNamee P, Soutter J, Wilsdon J, Matowe L, Needham G, Gilbert F, Bond S: **Effect of audit and feedback, and reminder messages on primary-care radiology referrals: a randomised trial.** *Lancet* 2001, **357:**1406-1409.

16. Emery J, Morris H, Goodchild R, Fanshawe T, Prevost AT, Bobrow M, Kinmonth AL: **The GRAIDS Trial: a cluster randomised controlled trial of computer decision support for the management of familial cancer risk in primary care.** *Br J Cancer* 2007, **97:**486-493.

17. Etter JF, Perneger TV: **Post-intervention effect of a computer tailored smoking cessation programme.** *J Epidemiol Community Health* 2004, **58:**849-851.

18. Feldstein A, Elmer PJ, Smith DH, Herson M, Orwoll E, Chen C, Aickin M, Swain MC: **Electronic medical record reminder improves osteoporosis management after a fracture: a randomized, controlled trial.** *J Am Geriatr Soc* 2006, **54:**450-457.

19. Filippi A, Sabatini A, Badioli L, Samani F, Mazzaglia G, Catapano A, Cricelli C: **Effects of an automated electronic reminder in changing the antiplatelet drug-prescribing behavior among Italian general practitioners in diabetic patients: an intervention trial.** *Diabetes Care* 2003, **26:**1497-1500.

20. Fox GN: **Electronic solutions to implementing lipid guidelines.** *J Fam Pract* 2002, **51:**872-874.

21. Fung CH: **Computerized condition-specific templates for improving care of geriatric syndromes in a primary care setting.** *J Gen Intern Med* 2006, **21:**989-994.

22. Frances CD, Alperin P, Adler JS, Grady D: **Does a fixed physician reminder system improve the care of patients with coronary artery disease? A randomized controlled trial.** *West J Med* 2001, **175:**165-166.

23. Fretheim A, Oxman AD, Håvelsrud K, Treweek S, Kristoffersen DT, Bjørndal A: **Rational prescribing in primary care (RaPP): a cluster randomized trial of a tailored intervention.** *PLoS Med* 2006, **3:**e134.

24. Glasgow RE, Nutting PA, King DK, Nelson CC, Cutter G, Gaglio B, Rahm AK, Whitesides H: **Randomized effectiveness trial of a computer-assisted intervention to improve diabetes care.** *Diabetes Care* 2005, **28:**33-39.

25. Goergen SK, Fong C, Dalziel K, Fennessy G: **Can an evidence-based guideline reduce unnecessary imaging of road trauma patients with cervical spine injury in the emergency department?** *Australas Radiol* 2006, **50:**563-569.

26. Goldberg HI, Neighbor WE, Cheadle AD, Ramsey SD, Diehr P, Gore E: **A controlled time-series trial of clinical reminders: using computerized firm systems to make quality improvement research a routine part of mainstream practice.** *Health Serv Res* 2000, **34:**1519-1534.

27. Goldberg HI, Neighbor WE, Hirsch IB, Cheadle AD, Ramsey SD, Gore E: **Evidence-based management: using serial firm trials to improve diabetes care quality.** *Jt Comm J Qual Improv* 2002, **28:**155-166.

28. Harpole LH, Khorasani R, Fiskio J, Kuperman GJ, Bates DW: **Automated evidence-based critiquing of orders for abdominal radiographs: impact on utilization and appropriateness.** *J Am Med Inform Assoc* 1997, **4:**511-521.

29. Hobbs FD, Delaney BC, Carson A, Kenkre JE: **A prospective controlled trial of computerized decision support for lipid management in primary care.** *Fam Pract* 1996, **13:**133-137.

30. Holbrook A, Keshavjee K, Lee H, Bernstein B, Chan D, Thabane L, Gerstein H, Troyan S, COMPETE II Investigators: **Individualized electronic decision support and reminders can improve diabetes care in the community.** *AMIA Annu Symp Proc* 2005:982.

31. Horowitz N, Moshkowitz M, Leshno M, Ribak J, Birkenfeld S, Kenet G, Halpern Z: **Clinical trial: Evaluation of a clinical decision-support model for upper abdominal complaints in primary-care practice.** *Aliment Pharmacol Ther* 2007, **26:**1277-1283.

32. Johnson SS, Driskell MM, Johnson JL, Dyment SJ, Prochaska JO, Prochaska JM, Bourne L: **Transtheoretical model intervention for adherence to lipid-lowering drugs.** *Dis Manag* 2006, **9:**102-114.

33. Johnson KB, Cowan J: **Clictate: a computer-based documentation tool for guideline-based care.** *J Med Syst* 2002, **26:**47-60.

34. Kashner TM, Rush AJ, Altshuler KZ: **Measuring costs of guideline-driven mental health care: the Texas Medication Algorithm Project.** *J Ment Health Policy Econ* 1999, **2:**111-121.

35. Keeffe B, Subramanian U, Tierney WM, Udris E, Willems J, McDonell M, Fihn SD: **Provider response to computer-based care suggestions for chronic heart failure.** *Med Care* 2005, **43:**461-465.

36. Kenealy T, Arroll B, Petrie KJ: **Patients and computers as reminders to screen for diabetes in family practice. Randomized-controlled trial.** *J Gen Intern Med* 2005, **20:**916-921.

37. Khoury A: **A computer-generated reminder program to reduce cardiac morbidity and mortality.** *Formulary* 1997, **32:**1241-1244.

38. Kitahata MM, Dillingham PW, Chaiyakunapruk N, Buskin SE, Jones JL, Harrington RD, Hooton TM, Holmes KK, University of Washington HIV Study Cohort: **Electronic human immunodeficiency virus (HIV) clinical reminder system improves adherence to practice guidelines among the University of Washington HIV Study Cohort.** *Clin Infect Dis* 2003, **36:**803-811.

39. Lesourd F, Avril C, Boujennah A, Parinaud J: **A computerized decision support system for ovarian stimulation by gonadotropins.** *Fertil Steril* 2002, **77:**456-460.

40. Lester WT, Grant RW, Barnett GO, Chueh HC: **Randomized controlled trial of an informatics-based intervention to increase statin prescription for secondary prevention of coronary disease.** *J Gen Intern Med* 2006, **21:**22-29.

41. Levenson D: **Reminders boost heart attack guideline compliance.** *Rep Med Guidel Outcomes Res* 2003, **14:**7-9.

42. Litzelman DK, Dittus RS, Miller ME, Tierney WM: **Requiring physicians to respond to computerized reminders improves their compliance with preventive care protocols.** *J Gen Intern Med* 1993, **8:**311-317.

43. Lobach DF, Hammond WE: **Computerized decision support based on a clinical practice guideline improves compliance with care standards.**  *Am J Med* 1997, **102:**89-98.

44. Lobach DF, Hammond WE: **Development and evaluation of a Computer-Assisted Management Protocol (CAMP): improved compliance with care guidelines for diabetes mellitus.** *Proc Annu Symp on Comput Appl Med Care* 1994:787-791.

45. Lobach DF: **Electronically distributed, computer-generated, individualized feedback enhances the use of a computerized practice guideline.** *Proc AMIA Annu Fall Symp* 1996:493-497.

46. Lorenzoni R, Ebert AG, Lattanzi F, Orsini E, Mazzoni A, Magnani M, Barbieri C, Rossi M, Mazzuoli F: **A computer protocol to evaluate subjects with chest pain in the emergency department: a multicenter study.** *J Cardiovasc Med (Hagerstown)* 2006, **7:**203-209.

47. McCartney P, Macdowall W, Thorogood M: **A randomised controlled trial of feedback to general practitioners of their prophylactic aspirin prescribing.** *BMJ* 1997, **315:**35-36.

48. McEwen A, West R, Preston A: **Triggering anti-smoking advice by GPs: mode of action of an intervention stimulating smoking cessation advice by GPs.** *Patient Educ Couns* 2006, **62:**89-94.

49. McMullin ST, Lonergan TP, Rynearson CS, Doerr TD, Veregge PA, Scanlan ES: **Impact of an evidence-based computerized decision support system on primary care prescription costs.** *Ann Fam Med* 2004, **2:**494-498.

50. Mitchell E, Sullivan F, Grimshaw JM, Donnan PT, Watt G: **Improving management of hypertension in general practice: a randomised controlled trial of feedback derived from electronic patient data.** *Br J Gen Pract* 2005, **55:**94-101.

51. Montori VM, Dinneen SF, Gorman CA, Zimmerman BR, Rissa RA, Bjornsen SS, Green EM, Bryant SC, Smith SA, Translation Project Investigator Group: **The impact of planned care and a diabetes electronic management system on community-based diabetes care.** *Diabetes Care* 2002, **25:**1952-1957.

52. Margolis CZ, Warshawsky SS, Goldman L, Dagan O, Wirtschafter D, Pliskin JS: **Computerized algorithms and pediatricians' management of common problems in a community clinic.** *Acad Med* 1992, **67:**282-284.

53. Morgan MM, Goodson J, Barnett GO: **Long-term changes in compliance with clinical guidelines through computer-based reminders.** *Proc AMIA Symp* 1998:493-497.

54. Nease J, Ruffin 4th. MT, Klinkman MS, Jimbo M, Braun TM, Underwood JM: **Impact of a generalizable reminder system on colorectal cancer screening in diverse primary care practices: a report from the prompting and reminding at encounters for prevention project.** *Med Care* 2008, **46:**S68-S73.

55. Nease DEJ, Green LA: **ClinfoTracker: a generalizable prompting tool for primary care.** *J Am Board Fam Pract* 2003, **16:**115-123.

56. Nilasena DS, Lincoln MJ: **A computer-generated reminder system improves physician compliance with diabetes preventive care guidelines.** *Proc Annu Symp Comput Appl Med Care* 1995:640-645.

57. Ornstein SM, Garr DR, Jenkins RG, Musham C, Hamadeh G, Lancaster C: **Implementation and evaluation of a computer-based preventive services system.** *Fam Med* 1995, **27:**260-266.

58. Palchuk MB, Seger DL, Alexeyev A, Macauley R, Seger AC, Recklet EG, Gandhi TK: **Implementing renal impairment and geriatric decision support in ambulatory e-prescribing.** *AMIA Annu Symp Proc* 2005:1071.

59. Pestotnik SL, Classen DC, Evans RS, Burke JP: **Implementing antibiotic practice guidelines through computer-assisted decision support: clinical and financial outcomes.** *Ann Intern Med* 1996, **124:**884-890.

60. Plaza V, Cobos A, Ignacio-García JM, Molina J, Bergoñón S, García-Alonso F, Espinosa C, Grupo IA: **[Cost-effectiveness of an intervention based on the Global INitiative for Asthma (GINA) recommendations using a computerized clinical decision support system: a physicians randomized trial].** *Medicina clínica* 2005, **124:**201-206.

61. Ramnarayan P, Roberts GC, Coren M, Nanduri V, Tomlinson A, Taylor PM, Wyatt JC, Britto JF: **Assessment of the potential impact of a reminder system on the reduction of diagnostic errors: a quasi-experimental study.** *BMC Med Inform Decis Mak* 2006, **6:**22.

62. Riddell T, Jackson RT, Wells S, Broad J, Bannink L: **Assessing Maori/non-Maori differences in cardiovascular disease risk and risk management in routine primary care practice using web-based clinical decision support: (PREDICT CVD-2).** *N Z Med J* 2007, **120:**U2445.

63. Rios M, Desandes E, Bresson B, Klein I, Lesur A, Boisson F, Demange V, Bey P: **[Clinical practice guidelines in cancerology: comparative study of three decision support-systems for breast and prostate cancer in Lorraine french region].** *Bull Cancer* 2003, **90:**363-370.

64. Rossi RA, Every NR: **A computerized intervention to decrease the use of calcium channel blockers in hypertension.** *J Gen Intern Med* 1997, **12:**672-678.

65. Roumie CL, Elasy TA, Greevy R, Griffin MR, Liu X, Stone WJ, Wallston KA, Dittus RS, Alvarez V, Cobb J, Speroff T: **Improving blood pressure control through provider education, provider alerts, and patient education: a cluster randomized trial.** *Ann Intern Med* 2006, **145:**165-175.

66. Safran C, Rind DM, Davis RB, Ives D, Sands DZ, Currier J, Slack WV, Cotton DJ, Makadon HJ: **Effects of a knowledge-based electronic patient record in adherence to practice guidelines.** *MD Comput* 1996, **13:**55-63.

67. Seroussi B, Bouaud J, Gligorov J, Uzan S: **Supporting multidisciplinary staff meetings for guideline-based breast cancer management: a study with OncoDoc2.** *AMIA Annu Symp Proc* 2007:656-660.

68. Shiffman RN, Freudigman KA, Brandt CA, Liaw Y, Navedo DD: **A guideline implementation system using handheld computers for office management of asthma: effects on adherence and patient outcomes.** *Pediatrics* 2000, **105:**767-773.

69. Smith MY, Cromwell J, DePue J, Spring B, Redd W, Unrod M: **Determining the cost-effectiveness of a computer-based smoking cessation intervention in primary care.** *Manag Care* 2007, **16:**48-55.

70. Subramanian U, Fihn SD, Weinberger M, Plue L, Smith FE, Udris EM, McDonell MB, Eckert GJ, Temkit M, Zhou XH, Chen L, Thierney WM: **A controlled trial of including symptom data in computer-based care suggestions for managing patients with chronic heart failure.** *Am J Med* 2004, **116:**375-384.

71. Thomas HV, Lewis G, Watson M, Bell T, Lyons I, Lloyd K, Weich S, Sharp D: **Computerised patient-specific guidelines for management of common mental disorders in primary care: a randomised controlled trial.** *Br J Gen Pract* 2004, **54:**832-837.

72. Tolman C, Richardson D, Bartlett C, Will E: **Structured conversion from thrice weekly to weekly erythropoietic regimens using a computerized decision-support system: a randomized clinical study.** *J Am Soc Nephrol* 2005, **16:**1463-1470.

73. Toth-Pal E, Nilsson GH, Furhoff AK: **Clinical effect of computer generated physician reminders in health screening in primary health care--a controlled clinical trial of preventive services among the elderly.** *Int J Med Inform* 2004, **73:**695-703.

74. van Steenkiste B, van der Weijden T, Stoffers HE, Kester AD, Timmermans DR, Grol R: **Improving cardiovascular risk management: a randomized, controlled trial on the effect of a decision support tool for patients and physicians.** *Eur J Cardiovasc Prev Rehabil*  2007, **14:**44-50.

75. van Wyk JT, van Wijk MA, Moorman PW, Mosseveld M, van der Lei J: **Cholgate - a randomized controlled trial comparing the effect of automated and on-demand decision support on the management of cardiovascular disease factors in primary care.** *AMIA Annu Symp Proc* 2003:1040.

76. Vissers MC, Biert J, van der Linden CJ, Hasman A: **Effects of a supportive protocol processing system (ProtoVIEW) on clinical behaviour of residents in the accident and emergency department.** *Comput Methods Programs Biomed* 1996, **49:**177-184.

77. Vissers MC, Hasman A, van der Linden CJ: **Impact of a protocol processing system (ProtoVIEW) on clinical behaviour of residents and treatment.** *Int J Biomed Comput* 1996, **42:**143-150.

78. Wells S, Furness S, Rafter N, Horn E, Whittaker R, Stewart A, Moodabe K, Roseman P, Selak V, Bramley D, Jackson R: **Integrated electronic decision support increases cardiovascular disease risk assessment four fold in routine primary care practice.** *Eur J Cardiovasc Prev Rehabil* 2008, **15:**173-178.

79. Westfall JM, Van Vorst RF, McGloin J, Selker HP: **Triage and diagnosis of chest pain in rural hospitals: implementation of the ACI-TIPI in the High Plains Research Network.** *Ann Fam Med* 2006, **4:**153-158.

80. Whitley HP, Fermo JD, Chumney EC: **5-year evaluation of electronic medical record flag alerts for patients warranting secondary prevention of coronary heart disease.** *Pharmacotherapy* 2006, **26:**682-688.

81. Ziemer DC, Tsui C, Caudle J, Barnes CS, Dames F, Phillips LS: **An informatics-supported intervention improves diabetes control in a primary care setting.** *AMIA Annu Symp Proc* 2006: 1160.
