# Supplementary material for: Effectiveness of electronic guideline-based implementation systems in ambulatory care settings - a systematic review
Source: Implement Sci. 2009 Dec 30;4:82. doi: 10.1186/1748-5908-4-82 (PMC2806389; doi:10.1186/1748-5908-4-82)
Supplement: Additional file 3 — Summary of Findings--expanded tables. Expanded versions of the tables with the summary of findings. Table 1 for comparison one, electronic multidimensional guidelines versus usual care and Table 2 and 3 for comparison two, electronic multidimensional guidelines versus another guideline implementation method. [file 1748-5908-4-82-S3.DOC]

**Table 1: Summary of Findings for comparison 1: Electronic multidimensional guidelines versus usual care**

IG = Intervention Group, CG = Control Group

| **Study** | **Risk of bias** | **No of patients, No of professionals** | **Intervention + implemented guideline** | **Outcomes** |
| --- | --- | --- | --- | --- |
|
| **Christakis [1]**  Cluster-RCT | low | 1339 visits for OM, 38 physicians | EB message system presenting real time evidence to providers based on their prescribing practice for otitis media.  Evidence-Based prescribing guidelines for  otitis media | Intervention providers had a 34% greater reduction in the proportion of time they prescribed antibiotics for <10 days (p=0.000). Intervention providers were less likely to prescribe antibiotics than were control providers (p=0.095). |
| **Davis [2]**  Cluster-RCT | low | 12195 visits, 44 health care providers | EB message system that presented real-time evidence to providers based on prescribing practices for acute otitis media, allergic rhinitis, sinusitis, constipation, pharyngitis, croup, urticaria, and bronchiolitis.  Evidence-Based recommendations for different pediatric diseases | Proportion of prescriptions dispensed in accordance with evidence improved significantly. The adjusted difference between the intervention and control groups was 8% (95% CI [1 - 15]). |
| **Meigs [3]**  Cluster-RCT | moderate | 598 patients, 66 health care providers | Diabetes Management Application, (DMA); interactive patient-specific clinical data, treatment advice and links to other web-based resources.  Guidelines for diabetes of the American Diabetes Association | Statistically significant as well as non-significant changes in processes and outcomes of care. Statistically significant increase in the number of HbA1c tests/year (p=0.008), the number of LDL cholesterol tests/year (p=0.02) and the proportion of patients undergoing at least one foot examination/year (p=0.003).  Variable use of the system (probably due to lack of integration in the workflow, time pressure and the belief that the use of computers during encounter is a barrier for effective communication) contributed to its relatively modest effects. |
| **Montgomery [4]**  Cluster-RCT | moderate | 614 patients, 27 general practices | Computer based clinical decision support system and a risk chart on absolute cardiovascular risk, blood pressure, and prescribing of cardiovascular drugs in hypertensive patients.  New Zealand guidelines for the management of hypertension | The results do not support the use of this computer based clinical decision support system in the management of hypertension. Patients in the computer based clinical decision support system were no more likely to have cardiovascular risk reduced to below 10% than patients receiving usual care. No significant differences in systolic and diastolic blood pressure and prescribing of cardiovascular drugs. |
| **Rollman [5]**  Cluster-RCT | moderate | 200 patients, 17 primary care physicians | Guideline-based treatment advice for depression:  Active care  Passive care  AHRQ Depression Panel's guideline | Patients’ mean HRS-D score decreased in all groups but neither continuous (p=0.8) nor categorical measures of recovery (p=0.2) differed between groups. Care processes for depression were also similar between groups, except for a statistically significant difference in the mean number of office visits with usual PCP at 6 months (p=0.02) and the % patients with $\geq$3 contacts with usual PCP at 6 months (p=0.03).  The reasons for the limited effect of the system were unclear, multiple causes (such as work overload during ambulatory visits and lack of patient participation) could be responsible according the authors. |
| **van Wyk [6]**  Cluster-RCT | moderate | 87866 patients, 77 primary care physicians | Clinical decision support system with respect to screening and treatment of dyslipidemia:  Alerting version  On-demand version  Guidelines of the Dutch College of General Practitioners for lipid management. | The alerting system improved screening (RR: 1.76; 95%CI [1.41-2.20]) and treatment performance (RR: 1.40; 95%CI [1.15-1.70]) of dyslipidemia compared with the usual care group. There was no statistically significant difference in screening and treatment of dyslipidemia between the on-demand system and the usual care group. |
| **Carton [7]**  ITS design | high | 6869 radiological examinations in sample | Reminder on screen indicating the appropriate recommendations concerning radiology requests  Guidelines written by CERF for radiology referral practice | Proportion of requests that did not conform to the guidelines was 33.2% when the guidelines were inactive and decreased to 26.9% when the recommendations were active (p 0.0001). More than 50% of abdominal plain radiographs requests, 24.9% of chest radiographs and 15.8% of CTs of the brain did not conform with recommendations.  The authors stated that practical impact of the intervention was rather poor possibly due to the limited intervention, neither feedback nor patient specific advice was provided. |
| **Day [8]**  ITS design | high | off: 103 patients  on: 258 patients  off: 125 patients | Real time advice regarding documentation, testing, treatment and disposition of emergency department patients with low back pain. (EDECS)  AHCPR guidelines for acute Low Back Pain | Significant improvement in documentation of the EMR and discharge instructions. Little effect on the appropriateness of testing and treatment and the cost of care. However EDECS was able to significantly decrease the number of patients who were told to stay in bed.  Failure to improve appropriateness of testing was probably reflective of the general confusion regarding the utility of plain X-rays in patients with low back pain. |
| **Hetlevik ’99 [9]**  Cluster-RCT | high | 2230 patients, 53 physicians | Clinical Decision Support System for hypertension  Norwegian clinical practice guidelines for hypertension | Patient outcomes (such as BMI, cholesterol levels, smoking habits, etc) and registrations were not affected in any clinically significant way. A statistically significant group difference in favour of the IG was observed in diastolic blood pressure (1 mmHg 95%CI [-1.89 - -0.17]), a significant baseline difference in systolic blood pressure in favour of the CG of 2.7 mmHg (95%CI [1.0 - 4.5]) had been reduced to 1.2 mmHg (95%CI [-0.6 - 3.0]) after intervention. |
| **Hetlevik ’00 [10]**  Cluster-RCT | high | 1034 patients, 53 physicians | Clinical Decision Support System for diabetes mellitus  Norwegian clinical practice guidelines for diabetes | The intervention did not result in a clinically significant change in doctors’ behaviour (registrations, prescribing of drugs and examinations) or in patient outcomes (HbA1c, blood pressure, serum cholesterol). A statistically significant difference was found in diastolic blood pressure in favour of the IG (-2.3 mmHg (95%CI [-3.8 - -0.8]).  Main problem seemed to be the content of the guidelines. The authors presumed that guidelines were rather seen as optional then as a standard, physicians are facing growing demands and time constraints, the intervention was time consuming and may have seemed unreasonable to perform from a practical and ethical point of view, lack of patients’ interest may have discouraged the physicians. |
| **Hicks [11]**  Cluster-RCT | high | 2027 patients, 14 primary care practices | Electronic decision support for hypertensive patients  National guidelines or standards were used including HEDIS, JNC VI and VI reports on the Prevention, Detection, Evaluation, and Treatment of High Blood Pressure and the AHA/ACC guidelines for cardiovascular disease prevention. | Increase in recommended medication prescribing compared to usual care (OR: 1.32; 95%CI [1.09-1.61]). No significant improvements, however, were observed in measures of blood pressure control (OR: 0.96; 95%CI [0.78-1.19]). |
| **McCowan [12]**  Cluster-RCT | high | 477 patients, 17 practice | Computerized decision support system for the management of asthma  Current British asthma clinical practice guidelines | No differences in reported symptoms, maintenance prescribing and use of hospital services between the two groups. A significant smaller proportion of patients within the IG initiated practice consultations for their asthma (OR=0.59, 95%CI [0.37 - 0.95]) and suffered acute asthma exacerbations (OR=0.43; 95%CI [0.21 - 0.85]).  Large number of dropouts because of technical problems and double data entry causing extra work overload. |
| **Poley [13]**  CBA design | high | 109 primary care physicians | Guideline-driven decision-support system for ordering blood tests in primary care  Clinical practice guidelines of the Dutch  College of General Practitioners for use of laboratory tests | The number of order forms submitted to the laboratories was not affected by the introduction of the CDSS (mean differences between changes: -7; 95% CI [-26 - 11]) but it did reduce the number of blood tests per order form statistically significant (mean differences between changes: -0.38; 95% CI [-0.61 - -0,16]) and yielded mean savings on the costs of laboratory requests of €847 per practice per 6 months (NS). |
| **Safran [14]**  CCT | high | 349 patients in analysis, 126 physicians and nurses | Reminders and alerts for HIV infection  Clinical practice guidelines developed by  expert panel HIV | No effect on hospitalisation or number of visits to the primary care practice. There was a significant increase in the rate of visits outside primary care (p=0.02), explained by the increased frequency of visits to ophthalmologists. There were no differences in survival (p=0.19). Effective in overall guideline compliance, the effect was more pronounced with alerts than with reminders. |
| **Schriger ’97 [15]**  ITS design |  | off: 50 patients  on: 156 patients  off: 74 patients | Real time advice regarding documentation, testing, treatment and disposition of emergency department patients regarding the management of body fluid exposure  (EDECS)  Existing CPG’s (literature review) for the management of body fluid exposure | Mean percent documentation of EMR and aftercare instructions, as well as appropriateness of testing and treatment increased statistically significant during the intervention phase while decreasing overall charges. All parameters returned to baseline when the system was removed. |
| **Schriger ’00 [16]**  ITS design | high | off: 352 patients  on: 374 patients  off: 104 patients | Real time advice regarding documentation, testing and treatment of children with fever presenting in the emergency department  (EDECS)  Existing CPG’s (literature review) for the management of fever in children | The intervention markedly improved documentation, had little effect on the appropriateness of the process of care and had no effect on charges.  Problems with diagnostic classification and low power may have contributed to its failure to modify test and treatment ordering behaviour, even as the controversy about the implemented guidelines. |
| **Sequist [17]**  Cluster-RCT | high | Diabetes: 4549 patients - CAD: 2199 patients, 194 physicians | Evidence-based electronic reminders for diabetes and Coronary Artery Disease  Evidence-Based Guidelines for Diabetes and Coronary Artery Disease | Electronic reminders increased the odds of overall recommended diabetes care (OR: 1.3; 95%CI [1.01-1.67]) and CAD (OR: 1.25; 95%CI [1.01-1.55]). The impact of individual reminders was variable, 3 of 9 reminders effectively increased rates of recommended care for diabetes or CAD.  Lack of time, the concurrent use of paper forms, deficiencies in documentation and lack of integration with computerized ordering likely limited the effect of the intervention. |
| **Szpunar [18]**  CBA design | high | Pre: 5334 patients – Post: 3970 patients, 6 clinics | Tobacco Use Cessation (TUC) Automated Clinical Practice Guideline  Tobacco Use Cessation CPG (a variation  of the US Department of Health and Human Services Clinical Practice Guideline on Treating Tobacco Use and Dependence) | Increases in guideline compliance for the 5 A’s in Tobacco Use Cessation, (ask, assess, advise, assist, and arrange). The differences among arms at post-implementation were statistically significant for ask, assess and arrange (p=0.001) and marginally significant for the assist guideline topic (p=0.07). |
| **Martens [19]**  Cluster-RCT | high | 53 primary care physicians | IG1: Reminders on antibiotics, asthma/COPD  IG2: Reminders on cholesterol lowering drugs  Prescription guidelines developed by a multidisciplinary committee. | No statistically significant differences between groups in sum scores per drug group (antibiotics, asthma/COPD and cholesterol) for all guideline recommendations.  Three individual volume measures had significant outcomes. Prescriptions of quinolones (*p* = 0.03), prescriptions of inhaled corticosteroids for newly diagnosed COPD patients older than 40 years (*p* = 0.00) and prescriptions of first choice drugs for acute sore throat (*p* = 0.03). |

**Table 2. Summary of Findings for comparison two: Electronic guideline implementations versus paper version of the guideline**

| **Study** | **Risk of bias** | **No of patients, No of professionals** | **Intervention** | **Outcomes** |
| --- | --- | --- | --- | --- |
|
| **Eccles [20]**  Cluster-RCT | low | 2400 patients, 60 primary care practices | IG1: Computerized asthma guidelines  + paper version of the guidelines for asthma and angina  IG2: Computerized angina guidelines  + paper version of the guidelines for asthma and angina  Evidence-Based clinical practice guidelines for asthma and angina | No effect was found on consultation rates, process of care measures (including prescribing, registrations and advice for examinations), or any patient reported outcomes for either condition.  Low levels of use were probably partly responsible for the lack of effect, staff had limiting training in functioning and use of the system and patients might not wish to discuss the problems even though the system might suggest it. |
| **Tierney ’03 [21]**  Cluster-RCT | low | 706 patients, 246 physicians | IG1: Computerized cardiac care suggestions + printed summary of the guidelines  IG2 and IG3: not included in analysis of this review  CG: Usual care + printed summary of the guidelines  AHCPR guidelines for managing heart disease adapted to local practice. | Care suggestions failed to improve adherence to guidelines or outcomes for patients with heart disease. Generic and condition-specific quality of life, acute exacerbations, medication compliance and healthcare costs were outcomes of interest.  Lack of physicians’ enthusiasm, their negative attitude towards CPG’s and the fact they probably found the intervention intrusive and time-consuming contributed to the failure of the system. |
| **Tierney ’05 [22]**  Cluster-RCT | low | 706 patients, 246 physicians | IG1: Computerized feedback for asthma and COPD + printed summary of the guidelines  IG2 and IG3: not included in analysis of this review  CG: Usual care + printed summary of the guidelines  Evidence-Based guidelines for managing  asthma and COPD (National Asthma Education Program Expert Panel Report. Executive Summary: Guidelines for the Diagnosis and Management of Asthma 1994 - Canadian Thoracic Society Workshop Group 1992) | Care suggestions had no effect on the delivery or outcomes of care for patients with reactive airway disease. Outcomes of interest were adherence to guidelines, health related quality of life and medication adherence.  Care suggestions were probably seen as nuisance which may have contributed to the failure of the system |
| **Jousimaa [23]**  Cluster-RCT | moderate | 2813 evaluated cases, 130 physicians | IG1: CD ROM of primary care guidelines  CG: Text based version of primary care guidelines  Collection of Finnish clinical practice guidelines to assist primary care physicians in daily practice decisions | No significant differences in all common elements of a physicians’ consultation between the computerized and textbook group, compliance rate was high in both groups. |
| **Montgomery [4]**  Cluster-RCT | moderate | 614 patients, 27 primary care practices | IG1: Computer based clinical decision support system and a risk chart on absolute cardiovascular risk, blood pressure, and prescribing of cardiovascular drugs in hypertensive patients.  IG2: Cardiovascular risk chart alone  CG: Usual care  New Zealand guidelines for the management of hypertension | Patients in the computer based clinical decision support system and chart only groups were no more likely to have cardiovascular risk reduced to below 10% than patients receiving usual care. Patients in the CDSS group were more likely to have a cardiovascular risk >10% than chart only patients (OR: 2.3; 95% CI[1.1 to 4.8]). No significant differences in systolic and diastolic blood pressure between the two groups. Patients in the chart only group were twice as likely to be prescribed two classes of cardiovascular drugs and over three times as likely to be prescribed three or more classes of drugs (p=0.0078).  One possible reason for the finding that the computer based clinical decision support system does not help manage cardiovascular risk is that the program may have distracted or confused the health professionals in their use of the risk chart. At the time of the study the computer based clinical decision support system was limited in the visual representation of the degree of risk. |
| **Murray [24]**  Cluster-RCT | moderate | 712 patients, 246 physicians | IG1: Computerized suggestions for hypertension + printed, referenced summary of the locally approved guidelines  IG2 and IG3: not included in analysis of this review  CG: Usual care + printed, referenced summary of the locally approved guidelines  Evidence-based clinical practice guidelines for uncomplicated hypertension published by the JNC. | The system failed to improve compliance with treatment suggestions or outcomes of patients with hypertension. Intergroup differences were neither statistically significant nor clinically relevant.  Negative findings could be explained by the potency of the system which was probably too weak, physicians received too many suggestions to comply with all of them, the suggestions might have been too complex and time-consuming. |
| **Wilson [25]**  Cluster-RCT | moderate | 86 practices | IG: Electronic referral guidelines for breast cancer + mailed referral guidelines  CG: Usual care + mailed referral guidelines  National guideline for cancer genetics | No statistically significant differences were observed between intervention and control arms in the primary or secondary outcomes  A possible reason for failure of the system could be the lack of integration with other practice systems. Low use of the system despite interactive workshops. |
| **Kuilboer [26]**  Cluster-RCT | high | 156772 patients, 40 primary care physicians | IG: AsthmaCritic provides patient-specific feedback for asthma and COPD + disposal of the asthma and COPD guidelines  CG: Usual care + disposal of the asthma and COPD guidelines  Clinical practice guidelines for asthma and COPD as issued by the Dutch College of General Practitioners | The manner in which the physicians monitored their patients changed, even as their treatment behaviour (e.g contact frequency, peak-flow and FEV1 measurements, prescribing). The change was lesser for their treatment behaviour as for monitoring and was only statistically significant for some outcomes (11 of 37 process measures) in some of the age groups. |

**Table 3. Summary of Findings for comparison two: Comparison of different types of electronic guideline implementation**

| **Study** | **Risk of bias** | **No of patients, No of professionals** | **Intervention** | **Outcomes** |
| --- | --- | --- | --- | --- |
|
| **Van Wijk [27]**  Cluster-RCT | low | 7094 patients,  44 practices | IG1: BloodLink Guideline  IG2: BloodLink Restricted  Clinical practice guidelines for blood tests ordering as issued by the Dutch College of General Practitioners | Decision support based on guidelines is more effective in changing blood test ordering than decision support based on initially displaying a limited number of tests. Primary care physicians who used BloodLink-Guideline requested 20% fewer tests on average than did practitioners who used BloodLink-Restricted (mean [±SD], 5.5 ± 0.9 tests vs 6.9 ± 1.6 tests (p=0.003)) |
| **Rollman [5]**  Cluster-RCT | moderate | 200 patients, 17 primary care physicians | IG1: Guideline-based treatment advice for depression: active care  IG2: Guideline-based treatment advice for depression: passive care  CG: Usual care  AHRQ Depression Panel's guideline | The guideline exposure condition had little differential impact on clinical outcomes or process measures. |
| **van Wyk [6]**  Cluster-RCT | moderate | 87866 patients, 77 primary care physicians | IG1: Clinical decision support system with respect to screening and treatment of dyslipidemia: alerting version  IG2: Clinical decision support system with respect to screening and treatment of dyslipidemia: on-demand version  CG: Usual care  Guidelines of the Dutch College of General Practitioners for lipid management. | The alerting version of the clinical decision support systems improved screening (RR: 1.40; 95%CI [1.08-1.81]). and treatment performance (RR: 1.18; 95%CI [0.96-1.45]) for dyslipidemia compared with the on-demand version. |

Reference List

1. Christakis DA, Zimmerman FJ, Wright JA, Garrison MM, Rivara FP, Davis RL: **A randomized controlled trial of point-of-care evidence to improve the antibiotic prescribing practices for otitis media in children.** *Pediatrics* 2001, **107:**E15.

2. Davis RL, Wright J, Chalmers F, Levenson L, Brown JC, Lozano P, Christakis DA: **A cluster randomized clinical trial to improve prescribing patterns in ambulatory pediatrics.** *PLoS Clin Trials* 2007, **2**:e25.

3. Meigs J, Cagliero E, Dubey A, Murphy-Sheehy P, Gildesgame C, Chueh H, Barry M, Singer D, Singer D, Nathan D**A: Controlled Trial of Web-Based Diabetes Disease Management: The MGH Diabetes Primary Care Improvement Project.** *Diabetes Care* 2003 **26**:750-757.

4. Montgomery AA, Fahey T, Peters TJ, MacIntosh C, Sharp DJ: **Evaluation of computer based clinical decision support system and risk chart for management of hypertension in primary care: randomised controlled trial.** *BMJ* 2000, **320:**686-690.

5. Rollman BL, Hanusa BH, Lowe HJ, Gilbert T, Kapoor WN, Schulberg HC: **A randomized trial using computerized decision support to improve treatment of major depression in primary care.** *J Gen Intern Med* 2002, **17:**493-503.

6. van Wyk JT, van Wijk MA, Sturkenboom MC, Mosseveld M, Moorman PW, van der Lei J: **Electronic alerts versus on-demand decision support to improve dyslipidemia treatment: a cluster randomized controlled trial.** *Circulation* 2008, **117:**371-378.

7. Carton M, Auvert B, Guerini H, Boulard JC, Heautot JF, Landre MF, Beauchet A, Sznajderi M, Brun-Ney D, Chagnon S: **Assessment of radiological referral practice and effect of computer-based guidelines on radiological requests in two emergency departments.** *Clin Radiol* 2002, **57:**123-128.

8. Day F, Hoang LP, Ouk S, Nagda S, Schriger DL: **The impact of a guideline-driven computer charting system on the emergency care of patients with acute low back pain.** *Proc Annu Symp Comput Appl Med Care* 1995:576-580.

9. Hetlevik I, Holmen J, Krüger O: **Implementing clinical guidelines in the treatment of hypertension in general practice. Evaluation of patient outcome related to implementation of a computer-based clinical decision support system.** *Scand J Prim Health Care* 1999, **17:**35-40.

10. Hetlevik I, Holmen J, Krüger O, Kristensen P, Iversen H, Furuseth K: **Implementing clinical guidelines in the treatment of diabetes mellitus in general practice. Evaluation of effort, process, and patient outcome related to implementation of a computer-based decision support system.** *Int J Technol Assess Health Care* 2000, **16:**210-227.

11. Hicks LS, Sequist TD, Ayanian JZ, Shaykevich S, Fairchild DG, Orav EJ, Bates DW: **Impact of computerized decision support on blood pressure management and control: a randomized controlled trial.** *J Gen Intern Med* 2008, **23:**429-441.

12. McCowan C, Neville RG, Ricketts IW, Warner FC, Hoskins G, Thomas GE: **Lessons from a randomized controlled trial designed to evaluate computer decision support software to improve the management of asthma.** *Med Inform Internet Med* 2001, **26:**191-201.

13. Poley MJ, Edelenbos K, I, Mosseveld M, van Wijk MA, de Bakker DH, van der Lei J, Rutten-van Mölken MP: **Cost consequences of implementing an electronic decision support system for ordering laboratory tests in primary care: evidence from a controlled prospective study in the Netherlands.** *Clin Chem* 2007, **53:**213-219.

14. Safran C, Rind DM, Davis RB, Ives D, Sands DZ, Currier J, Slack WV, Makadon HJ, Cotton DJ: **Guidelines for management of HIV infection with computer-based patient's record.** *Lancet* 1995, **346:**341-346.

15. Schriger DL, Baraff LJ, Rogers WH, Cretin S: **Implementation of clinical guidelines using a computer charting system. Effect on the initial care of health care workers exposed to body fluids.** *JAMA* 1997, **278:**1585-1590.

16. Schriger DL, Baraff LJ, Buller K, Shendrikar MA, Nagda S, Lin EJ, Mikulich VJ, Cretin S: **Implementation of clinical guidelines via a computer charting system: effect on the care of febrile children less than three years of age.** *J Am Med Inform Assoc* 2000, **7:**186-195.

17. Sequist TD, Gandhi TK, Karson AS, Fiskio JM, Bugbee D, Sperling M, Cook EF, Orav EJ, Fairchild DG, Bates DW: **A randomized trial of electronic clinical reminders to improve quality of care for diabetes and coronary artery disease.** *J Am Med Inform Assoc* 2005, **12:**431-437.

18. Szpunar SM, Williams PD, Dagroso D, Enberg RN, Chesney JD: **Effects of the tobacco use cessation automated clinical practice guideline.** *Am J Managed Care* 2006, **12:**665-673.

19. Martens JD, van der Weijden T, Severens JL, de Clercq PA, de Bruijn DP, Kester AD, Winkens RA: **The effect of computer reminders on GPs' prescribing behaviour: a cluster-randomised trial.** *Int J Med Inform* 2007, **76:**S403-S416.

20. Eccles M, McColl E, Steen N, Rousseau N, Grimshaw J, Parkin D, Purves I: **Effect of computerised evidence based guidelines on management of asthma and angina in adults in primary care: cluster randomised controlled trial.** *BMJ* 2002, **325:**941.

21. Tierney WM, Overhage JM, Murray MD, Harris LE, Zhou XH, Eckert GJ, Smith FE, Nienaber N, McDonald CJ, Wolinsky FD: **Effects of computerized guidelines for managing heart disease in primary care.** *J Gen Intern Med* 2003, **18:**967-976.

22. Tierney WM, Overhage JM, Murray MD, Harris LE, Zhou XH, Eckert GJ, Smith FE, Nienaber N, McDonald CJ, Wolinsky FD: **Can computer-generated evidence-based care suggestions enhance evidence-based management of asthma and chronic obstructive pulmonary disease? A randomized, controlled trial.** *Health Serv Res* 2005, **40:**477-497.

23. Jousimaa J, Mäkelä M, Kunnamo I, MacLennan G, Grimshaw JM: **Primary care guidelines on consultation practices: the effectiveness of computerized versus paper-based versions. A cluster randomized controlled trial among newly qualified primary care physicians.** *Int J Technol Assess Health Care* 2002, **18:**586-596.

24. Murray MD, Harris LE, Overhage JM, Zhou XH, Eckert GJ, Smith FE, Buchanan NN, Wolinsky FD, McDonald CJ, Tierney WM: **Failure of computerized treatment suggestions to improve health outcomes of outpatients with uncomplicated hypertension: results of a randomized controlled trial.** *Pharmacotherapy* 2004, **24:**324-337.

25. Wilson B, Torrance N, Mollison J, Wordsworth S, Gray J, Haites N, Grant A, Campbell M, Miedyzbrodzka Z, Clarke A, Watson M, Douglas A: **Improving the referral process for familial breast cancer genetic counselling: findings of three randomised controlled trials of two interventions.** *Health Technol Assess* 2005, **9**:iii-iv, 1-126.

26. Kuilboer M, van Wijk M, Mosseveld M, van der Does E, de Jongste J, Overbeek S, Ponsioen B, van der Lei J: **Computed critiquing integrated into daily clinical practice affects physicians' behavior--a randomized clinical trial with AsthmaCritic.** *Methods Inf Med* 2006, **45:**447-454.

27. van Wijk MA, van der LJ, Mosseveld M, Bohnen AM, van Bemmel JH: **Assessment of decision support for blood test ordering in primary care. a randomized trial.** *Ann Intern Med* 2001, **134:**274-281.
